# Supplementary material for: Complexome profiling of the Chlamydomonas psb28 mutant reveals TEF5 as an early PSII assembly factor
Source: Plant Cell. 2025 Mar 17;37(6):koaf055. doi: 10.1093/plcell/koaf055 (PMC12164590; doi:10.1093/plcell/koaf055)
Supplement: koaf055_Supplementary_Data [file koaf055_supplementary_data.zip › psb28_interactive.zip!/plots/intensity/qualityPlots/peptides/_Cre03.g201750.t1.2.html]

### Cre03.g201750.t1.2

Trivial name:   
SeqCoverage [median]: 14.40  
Mass: 27.96 kDa  
  
 

| Sequence | MissedCleavage | Mass [Da] | wtIntensity | muIntensity | Score |
| --- | --- | --- | --- | --- | --- |
| LKPEQYQAER | 1 | 1260.65 | 2169.84 | 3120.07 | 112.13 |
| LTEQEFR | 0 | 921.46 | 687.64 | 745.19 | 116.54 |
| NVVSLVEGAEEPEEEPAAAP | 0 | 2035.96 | 0.00 | 1150.52 | 103.97 |
|  |  |  | 2857.5 | 5015.8 |  |
